# The role of doxycycline in the therapy of multidrug-resistant *E. coli* – an *in vitro* study

**DOI:** 10.1038/srep31964

**Published:** 2016-08-18

**Authors:** Chih-Cheng Lai, Chi-Chung Chen, Hui-Ling Huang, Yin-Ching Chuang, Hung-Jen Tang

**Affiliations:** 1 Department of Intensive Care Medicine, Chi Mei Medical Center, Liouying, Tainan, Taiwan; 2 Department of Medical Research; Chi Mei Medical Center; Tainan, Taiwan; 3 Department of Health and Nutrition, Chia-Nan University of Pharmacy and Science, Tainan, Taiwan; 4 Department of Internal Medicine; Chi Mei Medical Center, Liouying, Tainan, Taiwan; 5 Department of Medicine, Chi Mei Medical Center, Tainan, Taiwan

## Abstract

This study assessed the *in vitro* antibacterial activity of combinations of amikacin and doxycycline or tigecycline against multidrug-resistant *E. coli* isolates. Twenty-four different pulsotypes, including 10 extended-spectrum β-lactamase (ESBL)-, 10 carbapenem-resistant, 2 New Delhi Metallo-beta-lactamase (NDM)- and 2 Klebsiella pneumoniae carbapenemase (KPC)-*E. coli* isolates were collected. All 24 isolates were susceptible to amikacin and tigecycline. Only 30% of ESBL and 50% of carbapenem-resistant *E. coli* were susceptible to doxycycline. Both of the NDM-*E. coli* had a MIC of 64 μg/ml. The checkerboard method showed that for the ESBL- and carbapenem-resistant *E. coli*, the synergistic effects of amikacin/doxycycline were 80% and 90%, respectively. For the two KPC- and two NDM-*E. coli*, the FIC index of amikacin/doxycycline were 0.5/0.375 and 0.5/0.281, respectively. For the ESBL- and carbapenem-resistant *E. coli* isolates, the combinations of amikacin and doxycycline exhibited synergistic activities against 80%, and 80% and 10% vs 60%, and 80% and 10% of the isolates at concentrations of 1x, 1/2x and 1/4xMIC, respectively. The synergistic effect seems to be similar for doxycycline and tigecycline based combinations with amikacin. In conclusion, the antibacterial activity of doxycycline can be enhanced by the addition of amikacin and is observed against most multidrug-resistant *E. coli* isolates.

*Escherichia coli*, a Gram-negative rod, can cause protean human infections, such as gastroenteritis, urinary tract infections, peritonitis, pneumonia and septicemia. The administration of appropriate antibiotics dependent upon susceptibility pattern is life-saving for the treatment of severe *E. coli* infections. However, the emergence of antibiotic-resistant *E. coli* has limited the therapeutic options available to physicians^1^,^2^. The production of β-lactamase is the most common mechanism of bacterial resistance to β-lactam. Among various antibiotic-resistant mechanisms, extended-spectrum β-lactamase (ESBL)- and carbapenemase-producing Enterobacteriaceae are spreading mostly as nosocomial pathogens worldwide, and the serious concern is that ESBL- and carbapenemase-producing Enterobacteriaceae are typically resistant to most of the currently available antibiotics^1^,^3^. To overcome this critical clinical condition, severe multi-drug resistant (MDR) bacterium infection, antimicrobial combination therapy with *in vitro* synergistic effect may be a better treatment option compared with monotherapy^4^,^5^,^6^,^7^,^8^. However, specific relevant investigations are scarce that guide the determination of the most appropriate combination antimicrobial therapy options.

Tigecycline, polymyxins, carbapenems, aminoglycosides, fluoroquinolones, fosfomycin, rifampicin, ampicillin-sulbactam, piperacillin-tazobactam, and tetracyclines (minocycline and doxycycline) are common antibiotics that have been used in combinations^9^,^10^,^11^. Although colistin, tigecycline, and some aminoglycosides remain most likely to be active *in vitro* against carbapenem-hydrolyzing β-lactamases producing Enterobacteriaceae, current data do not reliably support the use of these agents as monotherapy for systemic infections^2^. Antimicrobial combination therapy with these agents exhibiting synergistic effects might also be of benefit, and the investigation of further effective therapeutic regimens with various antibiotic combinations is warranted. However, most studies have been conducted to determine the *in vitro* activity of combination therapy against *Klebsiella pneumoniae* carbapenemase (KPC)-producing *Klebsiella pneumoniae*^4^,^5^,^6^,^7^. Enhanced activity was noted following treatment with doxycycline combined with amikacin against KPC-producing *K. pneumoniae* isolates in a recent study^7^; however, other *in vitro* studies of the combination effect of an aminoglycoside-amikacin with doxycycline or tigecycline against multi-drug resistant *E. coli* are rare. The goal of this study was to assess the *in vitro* antibacterial activity of the combinations of an aminoglycoside (amikacin) and doxycycline or tigecycline against ESBL-, carbapenem-resistant, New Delhi Metallo-beta-lactamase (NDM)- and KPC-producing *E. coli* isolates.

## Materials and Methods

### The collection of clinical isolates

Twenty-four different pulsotypes *E. coli* including 10 ESBL, 10 CRE, 2 KPC and 2 NDM strains were collected from the Department of Bacteriology at Chi Mei Medical Center between May 1, 2012 and April 30, 2014. Ethics approval was obtained from the Institution Review Board of the Chi Mei Medical Center. All of the methods were carried out in accordance with the relevant guidelines, and informed consent was obtained from all subjects. The isolates were stored at −80 °C in Protect Bacterial Preservers (Technical Service Consultants Limited, Heywood, UK) before use. ESBL was tested for use with both cefotaxime and ceftazidime, alone and in combination with clavulanic acid. An increase in the zone diameter of ≥5 mm for either antimicrobial agent tested in combination with clavulanic acid over that when tested alone indicates that the isolate is an ESBL producer^12^, excluding carbapenem resistant strains. Carbapenem resistance is defined as resistance to imipenem, meropenem, doripenem, or ertapenem. The carriage of KPC or NDM was confirmed by polymerase chain reaction (PCR) sequence analysis^13^. Species confirmation was performed by standard biochemical methods, on a VITEK 2 automated system (bioMérieux, Marcy l’Etoile, France).

### *In vitro* susceptibility

Standard powders of amikacin and doxycycline were obtained from Sigma, St Louis, MO. Tigecycline by Pfizer (New York, NY). MIC determinations and susceptibility interpretation criteria followed the CLSI and FDA standards^14^,^15^. The minimum inhibitory concentrations (MICs) of the drugs were measured by broth microdilution in freshly prepared Mueller-Hinton broth (Oxoid, Basingstoke, UK) with 25 μg/mL of calcium and 12.5 μg/mL of magnesium (CAMHB), as recommended by the CLSI guidelines^14^,^16^. *E. coli* ATCC 25922 was included as the control strain in each run of MIC measurements.

### The *in vitro* antibacterial activity of antibiotic combinations assessed by the broth method

The *in vitro* determination of the inhibitory effect of combination regimens followed the time-killing method was defined by the CLSI^17^. In brief, bacterial suspensions were diluted to concentrations 5.0×10^5^ colony-forming units (CFU)/mL in fresh Mueller–Hinton broth. Drug concentrations of amikacin, tigecycline and doxycycline were adjusted to those of 1xMIC, 1/2xMIC, and 1/4xMIC. Each drug alone and the combination of amikacin and tigecycline or doxycycline were tested. Bacterial counts were measured at 24 h by enumerating the colonies in 10-fold serially diluted specimens of 100 μL aliquots plated on the nutrient agar (Difco Laboratories, Sparks, MD) at 37 °C.

### Definitions 

Synergy was defined as a ≥2-log_10_ decrease in the CFU/ml between the combination and its most active constituent after 24 h and the number of surviving organisms in the presence of the combination must be ≥2 log_10_ CFU/ml below the starting inoculum. Bacteriostatic activities were defined as the presence of ≥2 log_10_, but <3 log_10_ reductions, and bactericidal activities were defined as the presence of ≥3 log_10_ reductions in the CFU/mL at 24 h, relative to the initial inoculum^17^. All experiments were performed in duplicate.

### The *in vitro* antibacterial activity of antibiotic combinations assessed by the checkerboard method

To evaluate the effect of the combinations, the fractional inhibitory concentration (FIC) was calculated for each combination by the broth microdilution technique as recommended by the CLSI and as previously described^14^,^18^,^19^. Briefly, the 96-well microdilution plates were inoculated with each test organism to yield the appropriate density (10^5 ^CFU/ml) in 100 μl of Mueller-Hinton broth (MHB) and incubated at 35 °C in ambient air for 24 h. One well with no antibiotic was used as a positive growth control on each plate. The plates were read for visual turbidity, and the results were recorded at 35 °C in ambient air using a magnifying mirror reader after 24 h of incubation, as turbidity in the wells indicated the growth of the microorganism. The MIC was determined as the well in the microtiter plate with the lowest drug concentration at which there was no visible growth. The following formulas were used to calculate the FIC index: FIC of drug A = MIC of drug A in combination/MIC of drug A alone, FIC of drug B = MIC of drug B in combination/MIC of drug B alone, and FIC index = FIC of drug A + FIC of drug B. Synergy was defined as a FIC index ≤0.5, indifference was defined as a FIC index >0.5 but ≤4, and antagonism was defined as a FIC index >4^20^. All drug combinations were performed repeatedly to validate the data.

### The detection of β-Lactamase genes

Plasmid DNA was extracted as templates and polymerase chain reaction (PCR) was used to amplify CTX-M, TEM, IMI, IMP, VIM, KPC, OXA and NDM using specific primers as previously published^21^,^22^,^23^. For AmpC genes, the following primers were used: (a) CMY-2-forward (TTT TCA AGA ATG CGC CAG GC), CMY-2-reverse (CTG CTG CTG ACA GCC TCT TT); and (b) DHA-1-forward (CTG ATG AAA AAA TCG TTA TC) and DHA-1-reverse (ATT CCA GTG CAC TCA AAA TA). For SHV genes, the following primers were used: (a) SHV-forward (GAT CCA CTA TCG CCA GCA GG) and SHV-reverse (ACC ACA ATG CGC TCT GC TTT G); and (b) SHV-12-forward (ATG CGT TAT ATT CGC CTG TG) and SHV-12-reverse (TTAGCGTTGCCAGTGCTCG). Amplicons were purified with PCR clean-up kits (Roche Diagnostics, GmbH, Penzberg, Germany) and were sequenced on an ABI PRISM3730 sequencer analyzer (Applied Biosystems, Foster City, CA, USA).

### Pulsed-field gel electrophoresis

PFGE was performed as described previously^24^ with a CHEF DR II apparatus (Bio-Rad Laboratories, Hercules, Calif.). In brief, the DNA in the plugs was digested with XbaI, and electrophoresis was performed in a 1% agarose gel (in 0.5x TBE [Tris-borate-EDTA] buffer). The electrophoretic conditions used were as follows: initial switch time, 2.0 s; final switch time, 35.0 s; run time, 21 h; gradient, 6 V/cm; angle, 120°; and temperature, 14 °C. The bacteriophage lambda ladder pulsed-field grade (PFG) and low-range PFG molecular weight markers were loaded onto all gels. The PFGE patterns were visually examined and interpreted according to the criteria of Tenover *et al*.^25^. The similarities of the PFGE profiles of each strain were compared using a Dice coefficient at 1.0% of tolerance and 0.8% of optimization.

## Results

**Figure 1:**
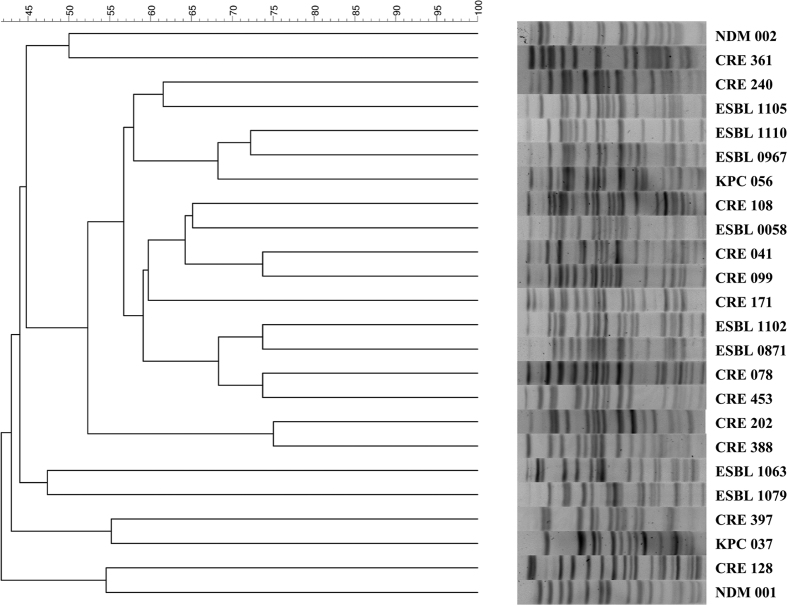
The PFGE profile of the enrolled 10 extended-spectrum β-lactamase (ESBL)-, 10 carbapenem-resistant-(CRE), 2 New Delhi Metallo-beta-lactamase (NDM)- and 2 Klebsiella pneumoniae carbapenemase (KPC)-producing *E. coli* isolates.

Figure 1 shows the PFGE profile of the enrolled 10 ESBL-, 10 carbapenem-resistant-, two KPC-2 producing and 2 NDM- *E. coli* isolates (one was NDM-1, and the other was NDM-5), and all of them had different PFGE profiles. Table 1 shows their MIC values and the susceptible rates of amikacin, doxycycline, and tigecycline. All of the 24 *E. coli* isolates were susceptible to amikacin and tigecycline. However, only 30% of ESBL *E. coli* and 50% of carbapenem-resistant *E. coli* were susceptible to doxycycline. For doxycycline, both of the NDM positive *E. coli* had MICs of 64 μg/ml, and in contrast, both KPC-producing *E. coli* had MIC values ≤ 2 μg/ml.

The ESBL and carbapenemase genes detected among the clinical isolates are presented in Table 2. For ten ESBL *E. coli* isolates, genes encoding CTX-M were detected for all isolates. Additionally, genes encoding TEM and CMY were detected for three and two isolates, respectively. For ten carbapenem-resistant *E. coli* isolates, genes encoding CMY were detected for all isolates. However, genes encoding CTX-M and TEM were detected for four and two isolates, respectively. For two KPC-producing *E. coli* isolates, genes encoding CMT, TEM, and CTX-M were detected for one isolate. For two NDM positive *E. coli* isolates, both had the gene encoding CMT and TEM, and one had the KPC-2 gene.

The results of the checkerboard methods are shown in Table 3. For the ESBL *E. coli*, the FIC_50/90_ of doxycycline and the tigecycline-based combination were 0.375/0.563 and 0.5/0.563, respectively. The synergistic effects of amikacin/doxycycline and amikacin/tigecycline were 80% and 60%, respectively. For carbapenem-resistant *E. coli*, the FIC_50/90_ of doxycycline and the tigecycline-based combination were 0.375/0.5, and 0.5/0.563, respectively. The synergistic effects of amikacin/doxycycline and amikacin/tigecycline were 90% and 80%, respectively. For both, there was no antagonism among the two combinations. For the two KPC *E. coli* and the two NDM *E. coli*, the FIC index values of amikacin/doxycycline were 0.5/0.375 and 0.5/0.281, respectively, and the FIC index values of amikacin/tigecycline were 0.375/0.5 and 0.265/0.312, respectively.

The *in vitro* activities of the combination of amikacin and doxycycline at the drug concentrations of 1xMIC, 1/2xMIC and 1/4x MIC against each isolate are shown in Table 4. For ESBL *E. coli*, the reduction of CFU at 24 hours ranged from 2.99 to 4.2, 0.05–4.2, and 0.29–4.08 log_10_, at concentrations of 1x, 1/2x and 1/4xMIC, respectively. The combinations of amikacin and doxycycline exhibited bactericidal effects against 90%, 70%, and 10% of the tested isolates at concentrations of 1x, 1/2x and 1/4xMIC, respectively. These combinations were synergistic against 80%, 80%, and 10% of the isolates at the concentrations of 1x, 1/2x and 1/4xMIC, respectively. For carbapenem-resistant *E. coli* isolates, the reduction of CFU at 24 hours ranged from 0.28 to 3.79, 0.23–3.53 and 0.73–2.00 log_10,_ at concentrations of 1x, 1/2x and 1/4xMIC, respectively. The combinations of amikacin and doxycycline exhibited bactericidal effects against 90%, 50%, and 10% of the tested isolates at concentrations of 1x, 1/2x and 1/4xMIC, respectively. These combinations were synergistic against 60%, 80%, and 10% of the isolates at concentrations of 1x, 1/2x and 1/4xMIC, respectively. For KPC *E. coli*, at the concentration of the 1x MIC combination, one of two strains had a synergistic effect, and the reduction of the CFU at 24 hours compared to the initial inoculum was 3.79 log_10_ and was −3.94 compared to most active antibiotic. At the concentration of 1/2x MIC, both strains had synergistic effects, and the reduction of the CFU at 24 hours compared to the initial inoculum was 3.79/2.10 log_10_ and was 6.45/4.49 compared to the most active antibiotic. Both strains had synergistic effects. The NDM strain combinations of amikacin and doxycycline were not performed because the MIC of doxycycline was too high.

The *in vitro* activities of combinations of amikacin and tigecycline at the drug concentrations of 1xMIC, 1/2xMIC and 1/4x MIC against each isolate are also shown in Table 4. For ESBL-*E. coli*, the reduction of CFU at 24 hours ranged from 2.18 to 3.72, 1.77–3.72 and 0.87–3.68 log_10,_ at concentrations of 1x, 1/2x and 1/4xMIC, respectively. The combinations of amikacin and tigecycline exhibited bactericidal effects against 90%, 90%, and 20% of the tested isolates at concentrations of 1x, 1/2x and 1/4xMIC, respectively. These combinations were synergistic against 50%, 100%, and 20% of the isolates at concentrations of 1x, 1/2x and 1/4xMIC, respectively. For carbapenem-resistant *E. coli*, the reduction of CFU at 24 hours ranged from 1.12 to 3.82, 2.00–3.82 and 0.30–1.56 log_10,_ at concentrations of 1x, 1/2x and 1/4xMIC, respectively. The combinations of amikacin and tigecycline exhibited bactericidal effects against 90%, 70%, and 0% of the tested isolates at concentrations of 1x, 1/2x and 1/4xMIC, respectively. These combinations were synergistic against 30%, 100%, and 0% of the isolates at concentrations of 1x, 1/2x and 1/4xMIC, respectively. For KPC *E. coli*, at the combined concentrations of 1x MIC and 1/4 x MIC, both two strains have no synergistic effect. At the combined concentration of 1/2x MIC, both strains had synergistic effects and with a value of 2.56/6.15 log_10_ compared to most active antibiotic. The reduction of CFU at 24 hours compared to the initial inoculum was 3.73/3.53 log_10_, exhibiting a bactericidal effect. One of the two NDM strains at the combined concentration of 1x MIC had a synergistic effect, and the reduction of CFU at 24 hours compared to the initial inoculum was 4.00 log_10_ was −2.38/0 log_10_ compares to the most active antibiotic. At the combined concentration of 1/2x MIC, both strains had a synergistic effect, and the reduction of CFU at 24 hours compared to the initial inoculum was 4.00/3.68 log_10_ and was 6.45/6.58 log_10_ compared to the most active antibiotic. However, no synergistic effect was noted at the combined 1/4 x MIC.

## Discussion

Antibiotic combination therapy has become the possible resolution for the treatment of severe multidrug resistant organism infections, and various antibiotic combination regimens for treating multidrug resistant *E. coli* have been recommended based on *in vitro* and *in vivo* studies. However, research investigating the *in vitro* antibacterial activity of the combinations of an aminoglycoside (amikacin) and tigecycline or doxycycline against multidrug-resistant *E. coli* isolates is scarce. This is the first study to assess this type of combined antibiotic regimen against multidrug-resistant *E. coli*, including ESBL-, carbapenem-resistant, NDM- and KPC-producing *E. coli* isolates. Based on this *in vitro* study, we have several significant findings. Most important, although tigecycline and amikacin displayed greater *in vitro* activities against multidrug-resistant *E. coli* than doxycycline, the synergistic effect seems to be similar between the combination of doxycycline and amikacin and the combination of tigecycline plus amikacin. As doxycycline is safe, inexpensive, and almost universally availability, further large *in vitro* and *in vivo* studies are warranted to clarify its role as a new adjunctive therapy to improve the outcomes of multidrug-resistant *E. coli* infections.

Although doxycycline is a cheap antimicrobial agent, it exhibits a broad spectrum of activity against different pathogens, including Gram-negative bacteria, and remains as a useful or even drug of choice in the treatment of many infectious diseases^26^,^27^. Even in this era of the increasing prevalence of multidrug-resistant organism infections, doxycycline is efficacious against multidrug-resistant *A. baumannii*^28^, *Pseudomonas aeruginosa*^29^, and *Stenotrophomonas maltophilia*^30^. In this first study investigating the *in vitro* activity of doxycycline against multidrug-resistant *E. coli*, we found that most clinical isolates, including seven (70%) ESBL-, five (50%) carbapenem-resistant and two (100%) NDM - *E. coli*, were not susceptible to doxycycline. However, even sub-inhibitory concentrations of an aminoglycoside combined with doxycycline can exhibit synergistic activities against more than 80% of tested isolates. For this combination, using 1/2xMIC of doxycycline (2 μg/mL, which is achievable in serum) produces the best synergism^7^,^31^. Therefore, our findings indicate the potential role of doxycycline-containing combinations in the management of multidrug-resistant *E. coli* infections.

Tigecycline, the first glycylcycline, exhibits potent activity against a wide range of clinically significant gram-positive and gram-negative bacteria, including multidrug-resistant strains (e.g., oxacillin-resistant *Staphylococcus aureus*, vancomycin-resistant enterococci, and ESBL-producing Enterobacteriaceae), and anaerobes (e.g., Bacteroides spp)^32^. Like several previous studies^33^,^34^,^35^, we found that the MIC values of tigecycline against all tested isolates remained low (≤1 μg/mL), and all of the tested isolates were susceptible to tigecycline. However, *in vitro* activity does not equate to an *in vivo* response, and the current suggested dosage of tigecycline for adults only achieves low serum concentrations; therefore, tigecycline cannot be recommend for the treatment of bloodstream infections, even those caused by so called “tigecycline-susceptible” isolates. To overcome this barrier to the treatment of critical conditions and the emergent tigecycline-resistant strains, tigecycline-containing combinations have been proposed as possible solutions. In this study, we found that using 1/2xMIC of tigecycline (0.5 μg/mL) in combination with sub-inhibitory concentrations of an aminoglycoside, synergism can be achieved for all of the 24 tested isolates. However, if we use 1/4xMICs of tigecycline (0.25 μg/mL) in combination with 1/4xMICs of an aminoglycoside, synergism was found for only two of the tested isolates. A previous study showed that the serum attainable concentration of tigecycline was only 0.38 and 0.93 μg/mL after a single dose injection of 50 mg and 100 mg tigecycline, respectively^36^. Therefore, if we formulate tigecycline-containing combination regimens based on the recommended dosages (100 mg loading, followed by 50 mg every 12 h), we can obtain the synergistic effect with tigecycline and amikacin despite low serum levels of tigecycline (<1 μg/mL).

In this study, we found an unusual association between NDM-1 and KPC-2 in one *E. coli* isolates, and it is the first detection of this combination in Taiwan. As previously reported^37^, this isolate should be multi-drug resistant against most antibiotics, excluding tigecycline. Previous studies only found this double carbapenemase-producer in *K. pneumoniae, E. cloacae, Citrobacter freundii* and *Enterobacter hormaechei* isolates from Brazil, Pakistan, China, and India^38^,^39^,^40^,^41^,^42^. However, we did not find the mutation of outer membrane porin (Omp) in KPC or NDM-producing isolates. As previous reports^43^,^44^,^45^, we found that the mutation of OmpA, OmpC, or OmpF was only presented in carbapenem-resistant strains. Overall, all of these findings indicate the worldwide emergence of double, or even multiple, carbapenemase-producing bacteria among Enterobacteriacae, including in Taiwan.

Finally, recent studies^46^,^47^ showed that the different resistance mechanisms of multidrug-resistant organisms may influence the synergistic effects of combination therapy. For carbapenem-resistant *K. pneumoniae*, Laishram *et al*.^46^ found that isolates producing NDM carbapenemase alone showed significantly more synergy than isolates producing OXA-48-like carbapenemase. Furthermore, Hong *et al*.^47^ found that clinical isolates of KPC-producing *K. pneumoniae* with high porin expression were more responsive to a combination of colistin-doripenm-ertapenem than isolates with low expression (100% [8/8] vs 0% [0/4]; p = 0.002). In this study of limited clinical isolates, we did not assess whether the MDR *E. coli* with different resistant mechanisms had different responses to antibiotic combination therapy. However, further investigations are warranted to clarify this issue.

In conclusion, despite the lower susceptible rate of doxycycline, the antibacterial activity of such an ancient antimicrobial agent can be enhanced by the addition of amikacin. The synergistic effect of such combinations seems to be as effective as the tigecycline/amikacin combination against most multidrug-resistant *E. coli* isolates, and warrants further *in vivo* investigation to confirm their therapeutic efficacy.

## Additional Information

**How to cite this article**: Lai, C.-C. *et al*. The role of doxycycline in the therapy of multidrug-resistant *E. coli* – an *in vitro* study. *Sci. Rep.* **6**, 31964; doi: 10.1038/srep31964 (2016).

## Figures and Tables

**Table 1:** The MIC values and susceptibility rates of amikacin, doxycycline, and tigecycline against 10 extended-spectrum β-lactamase (ESBL)-, 10 carbapenem-resistant-(CRE), 2 New Delhi Metallo-beta-lactamase (NDM)- and 2 Klebsiella pneumoniae carbapenemase (KPC)-producing *E. coli* isolates.

Antibiotics	ESBL (N=10)		CRE (N=10)		KPC		NDM		MIC breakpoint		
	MIC range	susceptible %	MIC range	susceptible %	KPC 037	KPC 056	NDM-1	NDM-2	S	I	R
Amikacin	2~16	100	2~16	100	8	2	8	2	≤16	32	≥64
Doxycycline	1~16	30	2~16	50	1	2	64	64	≤4	8	≥16
Tigecycline	0.12~1	100	0.5~1	100	0.5	0.25	1	0.25	≤2	4	≥8
Cefazolin	>128	0	>128	0	>128	>128	>128	>128	≦2	4	≧8
Cefmetazole	≦2~128	70	>128	0	8	4	>128	16	≦16	32	≧64
Cefotaxime	8~128	100	32~128	0	128	2	>128	>128	≦1	2	≧4
Cefpirome	≦2~16	30	≦2~128	20	4	≦2	128	16	≦2	4~8^a^	≧16
Doripenem	≦0.06	100	1~4	30	8	1	16	1	≦1	2	≧4
Ertapenem	≦0.06~0.12	100	4~64	0	8	32	32	8	≦0.5	1	≧2
Imipenem	0.25~1	100	2~16	0	8	4	64	8	≦1	2	≧4
Merapenem	≦0.06	100	1~4	20	8	2	16	2	≦1	2	≧4

^a^Susceptible-Dose Dependent (SDD).

**Table 2:** The extended-spectrum β-lactamase and carbapenemase genes detected among 10 extended-spectrum β-lactamase (ESBL)-, 10 carbapenem-resistant-(CRE), 2 New Delhi Metallo-beta-lactamase (NDM)- and 2 Klebsiella pneumoniae carbapenemase (KPC)-producing *E. coli* isolates.

isolates	CMY	TEM	CTX-M	KPC	NDM	others^a^	
ESBL *E. coli*							
ESBL 0041	2	1	14	—	—	—	
ESBL 0171	—	—	27	—	—	—	
ESBL 0871	—	—	14	—	—	—	
ESBL 0967	—	—	27	—	—	—	
ESBL 1063	—	—	24	—	—	—	
ESBL 1079	—	1	15	—	—	—	
ESBL 1102	2	—	15	—	—	—	
ESBL 1105	—	1	24	—	—	—	
ESBL 1110	—	—	174	—	—	—	
ESBL 0058	—	—	27	—	—	—	
CR *E. coli*							
CRE 078	2	—	—	—	—	—	
CRE 099	2	—	—	—	—	—	
CRE 108	2	—	15	—	—	—	
CRE 128	2	—	14	—	—	—	
CRE 202	2	—	—	—	—	—	
CRE 240	2	—	14	—	—	—	
CRE 361	2	1	—	—	—	—	
CRE 388	2	1	—	—	—	—	
CRE 397	42	—	14,15	—	—	—	
CRE 453	2	—	—	—	—	—	
KPC *E. coli*							
KPC 037	2^b^	1	3	KPC-2	—	—	
KPC 056	—	—	—	KPC-2	—	—	
NDM *E. coli*							
NDM 001	2	1	—	KPC-2	NDM-1	—	
NDM 002	2	1	—	—	NDM-5	—	

^a^Including SHV, DHA, VIM, IMP, OXA48.

^b^Insertion.

**Table 3:** The results of the checkerboard method of amikacin-based combinations with doxycycline and tigecycline against 10 extended-spectrum β-lactamase (ESBL)-, 10 carbapenem-resistant-(CRE), 2 New Delhi Metallo-beta-lactamase (NDM)- and 2 Klebsiella pneumoniae carbapenemase (KPC)-producing *E. coli* isolates.

	ESBL						CRE						KPC		NDM	
	range	FIC_50_	FIC_90_	S	I	A	range	FIC_50_	FIC_90_	S	I	A	FIC	FIC	FIC	FIC
Amikacin/Doxycycline	0.25~0.625	0.375	0.563	80	20	0	0.25~0.563	0.375	0.5	90	10	0	0.5	0.375	0.5	0.281
Amikacin/Tigecycline	0.31~0.75	0.5	0.563	60	40	0	0.375~0.563	0.5	0.563	80	20	0	0.375	0.5	0.265	0.312

S, I, R: Synergy (%), Indifference (%), Antagonism (%).

**Table 4:** The log change (log_10_ CFU/ml) from the starting inoculum and the most active single agent after 24 h of incubation with different concentrations of antibiotics combinations including 1x, 1/2x and 1/4x MICs of amikacin, doxycycline and tigecycline for 10 extended-spectrum β-lactamase (ESBL)-, 10 carbapenem-resistant-, 2 New Delhi Metallo-beta-lactamase (NDM)- and 2 Klebsiella pneumoniae carbapenemase (KPC)-producing *E. coli* isolates.

(a) ESBL												Synergism(%)	-cidal/ -static(%)^a^
		Colony changes (log_10 _CFU/mL) at 24 h											
		ESBL0041	ESBL0058	ESBL0171	ESBL0871	ESBL0967	ESBL1063	ESBL1079	ESBL1102	ESBL1105	ESBL1110		
1xAMK+1xDOX	vs. initial inoculum	−3.76	−4.08	−3.73	−3.82	−4.20	−3.88	−3.79	−2.99	−3.51	−3.73	80	90/10
	vs. most active drug	−2.51	0.00	−5.83	−3.81	−2.66	−3.44	−1.73	−2.90	−3.93	−4.90		
1/2xAMK+1/2xDOX	vs. initial inoculum	−3.76	−4.08	0.82	−2.62	−4.20	−3.88	−3.79	−0.05	−3.51	−3.73	80	70/10
	vs. most active drug	−5.83	−6.81	−2.22	−5.00	−6.95	−6.15	−6.34	−2.98	−5.92	−6.76		
1/4xAMK+1/4xDOX	vs. initial inoculum	0.00	−4.08	3.09	1.60	−0.54	0.06	1.76	3.24	−1.51	−0.29	10	10/0
	vs. most active drug	−2.94	−6.70	0.48	−1.59	−3.07	−1.66	−1.32	0.00	−5.00	−3.55		
1xAMK+1xTGC	vs. initial inoculum	−3.64	−3.56	−3.60	−3.51	−3.51	−3.70	−3.45	−2.18	−3.68	−3.72	50	90/10
	vs. most active drug	−2.60	−0.9	−2.53	−2.34	−1.00	0.00	−4.87	1.51	−1.83	−3.34		
1/2xAMK+1/2xTGC	vs. initial inoculum	−3.64	−3.56	−3.60	−3.51	−3.51	−3.70	−2.07	−3.68	−3.68	−3.72	100	90/10
	vs. most active drug	−5.95	−5.90	−5.86	−7.00	−6.34	−5.90	−5.12	−4.86	−3.51	−3.81		
1/4xAMK+1/4xTGC	vs. initial inoculum	3.16	3.44	−0.87	3.21	−0.64	0.78	3.25	−1.27	−3.68	−3.41	20	20/0
	vs. most active drug	0.16	0.55	−4.24	0.30	−3.69	−1.97	0.80	−4.33	−6.41	−6.54		
(b) CRE													
		Colony changes (log_10_ CFU/mL) at 24 h										Synergism(%)	-cidal/ -static(%)^a^
		CRE078	CRE099	CRE108	CRE128	CRE202	CRE240	CRE361	CRE388	CRE397	CRE453		
1xAMK+1xDOX	vs. initial inoculum	−3.53	−3.51	−3.48	−3.78	−3.51	−3.15	−3.79	−3.76	−3.00	−0.28	60	90/0
	vs. most active drug	0.00	−2.78	−2.56	0.00	−1.45	−5.04	−3.99	−3.60	−3.54	−0.09		
1/2xAMK+1/2xDOX	vs. initial inoculum	−3.53	−2.73	−2.33	−0.78	−3.51	−3.45	−3.01	−3.46	−2.15	−0.23	80	50/30
	vs. most active drug	−5.41	−4.12	−5.13	−2.45	−6.20	−5.15	−5.56	−5.68	−5.55	−3.70		
1/4xAMK+1/4xDOX	vs. initial inoculum	−0.73	2.05	2.7	2.73	3.35	1.97	1.92	−1.03	−2.00	3.47	10	0/10
	vs. most active drug	−1.57	−1.44	−0.37	−0.45	0.44	−0.38	0.92	−1.71	−5.28	0.00		
1xAMK+1xTGC	vs. initial inoculum	−3.53	−3.20	−3.82	−3.68	−3.51	−3.45	−3.79	−3.76	−3.60	−1.12	30	90/0
	vs. most active drug	0.00	−2.49	−0.30	−1.89	−1.45	−1.08	−2.56	−2.90	−0.78	−2.84		
1/2xAMK+1/2xTGC	vs. initial inoculum	−3.53	−2.00	−3.82	−3.68	−3.51	−2.54	−3.79	−3.76	−3.60	−2.45	100	70/30
	vs. most active drug	−7.00	−5.09	−3.79	6.34	−6.38	−4.24	−5.51	−3.66	−5.95	−5.80		
1/4xAMK+1/4xTGC	vs. initial inoculum	1.61	3.3	−1.56	2.02	1.70	3.13	2.76	2.74	−0.30	3.17	0	0/0
	vs. most active drug	0.77	−0.14	−4.12	−0.16	−1.05	0.79	1.76	2.06	−3.62	0.04		
(c) KPC/NDM													
		KPC						NDM					
		Colony changes (log_10_ CFU/mL) at 24 h				synergism (%)	-cidal/-static (%)^a^	Colony changes (log_10_ CFU/mL) at 24 h				synergism (%)	-cidal/-static (%)^a^
		KPC 037		KPC 056				NDM 001		NDM 002			
1xAMK+1xDOX	vs. initial inoculum	−3.79		−3.79		50	100/0	ND		ND		ND	ND
	vs. most active drug	−0.30		−3.94				ND		ND			
1/2xAMK+1/2xDOX	vs. initial inoculum	−3.79		−2.10		100	50/50	ND		ND		ND	ND
	vs. most active drug	−6.45		−4.49				ND		ND			
1/4xAMK+1/4xDOX	vs. initial inoculum	3.14		1.79		0	0/0	ND		ND		ND	ND
	vs. most active drug	−0.01		−1.18				ND		ND			
1xAMK+1xTGC	vs. initial inoculum	−3.73		−3.53		0	100/0	−4.00		−3.68		50	100/0
	vs. most active drug	−1.82		−1.58				−2.38		0.00			
1/2xAMK+1/2xTGC	vs. initial inoculum	−3.73		−3.53		100	100/0	−4.00		−3.68		100	100/0
	vs. most active drug	−2.56		−6.15				−6.45		−6.68			
1/4xAMK+1/4xTGC	vs. initial inoculum	3.00		−0.33		0	0/0	2.68		−0.93		0	0/0
	vs. most active drug	0.18		−3.53				0.10		−3.90			

-cidal refers to the bactericidal effect and -static refers to the bacteriostatic effect. ND refers to not done.

^a^-cidal refers to bactericidal effect and -static refers to bacteriostatic effect. ND refers to not done.
